# Dual role of iodine, silver, chlorhexidine and octenidine as antimicrobial and antiprotease agents

**DOI:** 10.1371/journal.pone.0211055

**Published:** 2019-01-31

**Authors:** Vojtěch Pavlík, Martin Sojka, Mária Mazúrová, Vladimír Velebný

**Affiliations:** 1 Cell Physiology Research Group, Contipro, Dolní Dobrouč, Czech Republic; 2 Institute of Dermatology, 3rd Medical Faculty, Charles University in Prague, Prague, Czech Republic; 3 Institute of Microbiology, Faculty of Medicine, Slovak Medical University, Bratislava, Slovakia; NYU Langone Medical Center, UNITED STATES

## Abstract

**Objectives:**

The majority of human chronic wounds contain bacterial biofilms, which produce proteases and retard the resolution of inflammation. This in turn leads to elevated patient protease activity. Chronic wounds progressing towards closure show a reduction in proteolytic degradation. Therefore, the modulation of protease activity may lead to the faster healing of chronic wounds. Antimicrobials are used to control biofilm-based infection; however, some of them also exhibit the inhibition of matrix metalloproteinases and bacterial proteases. We investigated the antimicrobial agents used in wound healing for their potential to inhibit bacterial and host proteases relevant to chronic wounds.

**Methods:**

Using *in vitro* zymography, we tested the ability of povidone-iodine, silver lactate, chlorhexidine digluconate, and octenidine hydrochloride to inhibit selected human proteases and proteases from *Pseudomonas aeruginosa*, *Staphylococcus aureus*, *Serratia marcescens*, and *Serratia liquefaciens*. We investigated penetration and skin protease inhibition by means of *in situ* zymography.

**Results:**

All the tested antimicrobials inhibited both eukaryotic and prokaryotic proteases in a dose-dependent manner *in vitro*. The tested compounds were also able to penetrate into skin *ex vivo* and inhibit the resident proteases. Silver lactate and chlorhexidine digluconate showed an inhibitory effect *ex vivo* even in partial contact with skin in Franz diffusion cells.

**Conclusions:**

Our *in vitro* and *ex vivo* results suggest that wound healing devices which contain iodine, silver, chlorhexidine, and octenidine may add value to the antibacterial effect and also aid in chronic wound healing. Antiprotease effects should be considered in the design of future antimicrobial wound healing devices.

## Introduction

The healing of chronic wounds is complicated among other factors by the elevated expression and activity of matrix metalloproteases (MMPs), which need to decrease in amount or activity to aid healing progression [1]. In addition to these host proteases, biofilm-forming bacteria also produce their respective proteases that act in synergy with the abundant human proteases and contribute to the degradation of newly formed granulation tissue and growth factors [2]. This highlights the importance of regulated proteolysis in wound closure. Due to the severity of excessive protease activity, a subset of advanced wound dressings has been developed to decrease the activity of host metalloproteinases and serine proteases, usually by nonspecific absorption into the bandage [3] or by incorporating alternative “bait” collagen substrate [4]. However, the antibacterial treatment of infected wounds must also be maintained.

In this respect, some antimicrobials offer the combined action of antibacterial and antiprotease effects. Iodine solutions have been shown to inhibit proteases in wound exudates [5] or proteases from other sources [6], while chlorhexidine has been shown to decrease the activity of MMPs and bacterial proteases [7,8]. Silver compounds have exhibited inhibitory effects on MMPs and non-eukaryotic proteases [6,9].

Generally, there is little or no information on the ability of antimicrobials to inhibit proteases, especially in the context of wound proteases. Yet, at least some antimicrobials could help to break the vicious cycle of elevated chronic wound proteases and may therefore be repurposed to fulfil dual roles in wound healing.

We hypothesised that antimicrobials used in wound healing can also reduce the activity of wound proteases. We evaluated povidone iodine, silver lactate, chlorhexidine digluconate, and octenidine hydrochloride to see whether they could inhibit proteases from human leukocytes or human pathogenic bacteria *in vitro* and also whether such antimicrobials could decrease the activity *of* endogenous proteases in porcine skin *ex vivo*. Thus, the antimicrobial inhibitory action was tested with proteases relevant to human chronic wounds.

## Materials and methods

### Sources of proteases

*Pseudomonas aeruginosa*, *Staphylococcus aureus*, *Serratia marcescens*, and *Serratia liquefaciens* were isolated from human chronic ulcers as described previously [10]. The bacteria were inoculated into a Bolton broth base (Merck Life Science, Germany) with 2% gelatin (Thermo Fisher Scientific, USA) and grown overnight (37°C, 150 rpm). The resulting cultures were freeze-thawed twice and then centrifuged to obtain a supernatant containing bacterial proteases. Human neutrophils were isolated from peripheral blood from donors using Ficoll-Paque (Merck Life Science, Germany) according to the manufacturer’s protocol, then disrupted with 2 freeze-thaw cycles and centrifuged to collect supernatant; all donors gave their informed consent. By means of the migration method, primary keratinocytes and fibroblasts were isolated from eyelids that were removed during routine plastic surgeries (Galen, Ústí and Orlicí, Czech Republic); again, donors gave their informed consent. Fibroblasts were cultivated in Dulbecco’s modified Eagles medium–low glucose (DMEM) supplemented with 10% FBS, glutamine (0.3 mg.mL^-1^), glucose (4 mg.mL^-1^), penicillin (100 units.mL^-1^) and streptomycin (0.1 mg.mL^-1^)–in 5% CO_2_ at 37°C in a 6 well cultivation panel until the fifth passage. Keratinocytes were cultivated in the same way but without the addition of glucose to the medium. The induction of MMPs from fibroblasts and keratinocytes was performed using a 60 mJ.cm^-2^ UV-B simulator (Oriel Instruments, Newport, NY, USA). The conditioned serum-free medium was collected 24 hours after irradiation. Trypsin used for the fluorogenic assay was diluted from stock solution (Trypsin-EDTA Solution 10X, Merck Life Science, Germany). Human recombinant MMP-2 expressed in *E*. *coli* was purchased from Merck Life Science (Germany).

Eukaryotic and bacterial culture media were tested for gelatinase activity using gelatin zymography. Samples with proteases from fibroblasts, keratinocytes, and neutrophils were mixed together so that the gelatinolytic activities of the mixed components were similar. This was also done for prokaryotic proteases. The overall gelatinolytic activity of protease mixes corresponded to 15 pg of human recombinant MMP-2 (Merck Life Science, Germany).

### Antimicrobials

Povidone-iodine (PVP-I, Betadine, EGIS Pharmaceuticals PLC, Hungary) stock solution contained 100 mg.mL^-1^ of PVP-I, from which there was 10 mg.mL^-1^ of active substance. Silver lactate (Merck Life Science, Germany) was dissolved in demineralized water to a concentration of 70 mg.mL^-1^ prior to use and shielded from direct light and any contact with metals. Chlorhexidine digluconate was diluted directly into the assay buffer from the stock solution (20% aqueous solution, Merck Life Science, Germany). Octenidine hydrochloride (Dishman Pharma & Chem.Ltd, India) was initially dissolved to a 3% solution in 96% ethanol.

### Gelatin zymography

Standard SDS-PAGE was performed using 10% acrylamide gel with 0.2% gelatin [11]. The washing step in 2.5% Triton X-100 (Merck Life Science, Germany) was extended to 45 mins with three buffer exchanges. The incubation in development buffer (50 mM HEPES with 5 mM calcium nitrate, pH 7.8) lasted 17 hours at 37°C. We did not use the usual Tris-HCl development buffer, because chloride ions precipitate chlorhexidine and silver. Two pairs of prokaryotic and eukaryotic protease mixes were loaded on each 1 mm gel and run at 120 V for 90 min (Mini-Protean Tetra Cell, Bio-Rad, CA, USA). The gels were cut into halves after electrophoresis, placed into separate Petri dishes, and incubated in antimicrobial or control developing solution. PVP-I was diluted directly from the stock solution to yield concentrations of 10, 2, and 0.4 mg.mL^-1^. Silver lactate was tested in the concentration series 7, 1.4, and 0.28 mg.mL^-1^. Chlorhexidine digluconate was used in incubation at concentrations of 0.5 and 0.1 mg.mL^-1^, and the concentration of octenidine hydrochloride in the development buffer was 100, 20, or 4 μg.mL^-1^. Each concentration was tested in four independent replicates.

The developed gels were stained with Coomassie Brilliant Blue solution and photographed, and the zymographic bands were analysed using densitometry in Fiji software [12]. The computed densities of the bands for each treatment (n = 4) were related to the untreated control (n = 4). The normality of the sample distribution was tested with the Shapiro-Wilk test in R [13]. Where normality was not rejected, the significance of the change in protease activity was compared to control using Student’s one sample t-test (Excel, Microsoft Office).

### Fluorogenic zymography

DQ gelatin (ThermoFisher Scientific, USA) was dissolved to a concentration of 1 mg.mL^-1^ in demineralized water, desalted using ultracentrifugation (3 500 kDa MWCO), as the salt would induce silver to precipitate, and stored at 4°C. The DQ gelatin assay concentration was constant for all measurements (40 μg/reaction). Different buffers were needed for each tested compound to remain in solution. Chlorhexidine was diluted in a buffer (50 mM HEPES, 10 mM Ca(NO_3_)_2_, 1 μM ZnSO_4_, pH 6.9) in a two-fold dilution series starting from 2.5% (final assay concentration, 1%). Silver lactate was diluted in a buffer (50 mM HEPES, 10 mM Ca(NO_3_)_2_, 1 μM ZnSO_4_, pH 6.0) in a two-fold series starting at 25 mg.mL^-1^ (final concentration, 10 mg.mL^-1^). Octenidine hydrochloride was diluted in a buffer (50 mM HEPES, 1 μM ZnSO_4_, pH 6.9) in a two-fold series starting from 75 μg.mL^-1^ (final concentration, 30 μg.mL^-1^). Iodine quenches fluorescence and therefore is not suitable for this assay. All sources of proteases (either pure or from cultivation media/lysate) were also desalted by means of ultracentrifugation (3 500 kDa MWCO). As we kept the substrate reaction concentration constant, we diluted the proteases to prevent limitation of the reaction rate by the substrate concentration. The proteases had similar activity. The total protein concentration in all reactions was adjusted to 0.3 mg.mL^-1^ with BSA. Each assay contained 40 μL of diluted antimicrobial agent in the specified buffer, 10 μL protease(s), and 50 μL DQ gelatin diluted in the same buffer. Protease activity was measured (Ex 485 nm, Em 520 nm, EnSight, Perkin-Elmer, Waltham, MA, USA) continuously for 150 minutes. The increase in fluorescence between 10 and 30 min from the start of the reaction was linear and therefore the proteases were not limited by the substrate. We measured seven concentrations for each antimicrobial and buffer-only control reaction. The measurements for each condition were repeated two to three times. Differences in fluorescence increase between treated and control samples were expressed as percentage of activity. These differences were fitted using four-parameter log-linear curves and plotted in the R statistical environment [13] with functions provided from the drc package [14], which was also used for the estimation of IC50 (0.95 CI estimation with delta type confidence interval).

### Porcine dermis

Porcine skin was excised from the inner parts of ears obtained from a slaughterhouse (Bocus, Letohrad, Czech Republic). The epidermis was peeled off after 2 min immersion in a 60°C water bath. The dermis was either incubated directly in tubes with the tested compounds or partially exposed to antimicrobials in Franz diffusion cells [15] for 24 hours. A commercial inhibitor of proteases (Pierce Protease Inhibitor Mini Tablets, ThermoFisher Scientific, USA) was also tested on skin samples.

Dermis was washed thoroughly in PBS after the incubation in tubes and disintegrated with TissueLyser II (Qiagen, Germany) by means of bead beating (20 min, 30 Hz). The homogenate was centrifuged (10 min, 10 000 g) and the protein concentration was determined (Pierce BCA Protein Assay Kit, ThermoFisher Scientific, USA). Samples diluted to the same protein concentrations were analysed by means of gelatin zymography.

### In situ zymography

The samples of porcine dermis incubated in Franz diffusion cells with antimicrobials and protease inhibitors were rinsed in PBS, fixed in zinc buffer, and processed as described [16]. Cryosections were incubated with DQ gelatin fluorogenic substrate at 37°C or at -20°C (nonspecific background control) for 1 hr. Afterwards, the slides were scanned (Eclipse-Ti, Nikon, Japan). The fluorescence intensity in a square of standardized dimensions was evaluated in Fiji Software. The mean fluorescent intensity for each treatment (n = 4) was compared to the PBS-treated controls (n = 4). The normality of the sample distribution was evaluated using the Shapiro-Wilk test in R [13]. Where normality was not rejected, the statistical significance of changes in fluorescence was compared with Student’s one sample t-test with null hypothesis assuming the sample mean to be equal to 100% (control) (Excel, Microsoft Office).

## Results

**Fig 1:**
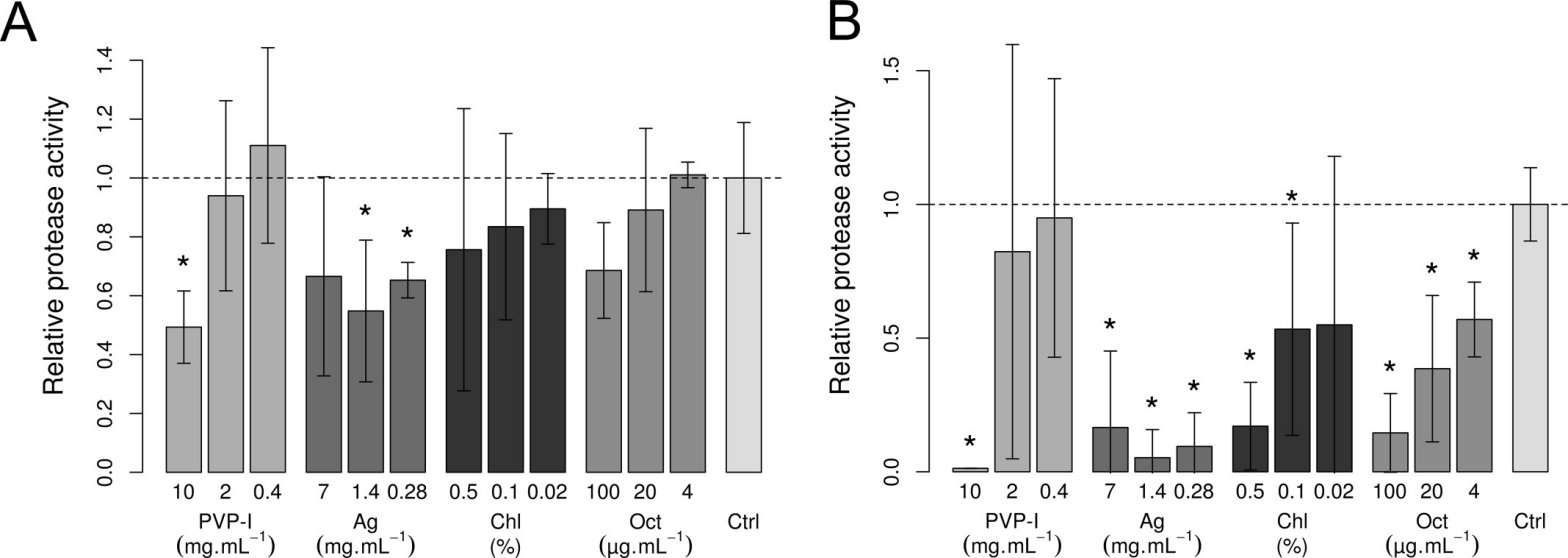
Activity of bacterial proteases with antimicrobials in SDS-PAGE zymography. (A) A mixture of bacterial proteases was separated on polyacrylamide gels with gelatin and incubated overnight in developing buffer that contained decreasing amounts of povidone-iodine (PVP-I), silver lactate (Ag), chlorhexidine digluconate (Chl), and octenidine hydrochloride (Oct). The values are expressed as relative protease activity related to control (Ctrl) with no added antimicrobial. (B) The activity of eukaryotic proteases similarly incubated with varying concentrations of antimicrobials. In panels A and B, bars represent standard deviation, * denotes p < 0.05 (Student's one sample t-test to control), n = 4.

We tested whether a dilution series of povidone-iodine (PVP-I), silver lactate, chlorhexidine digluconate, and octenidine hydrochloride can hinder the activity of mammalian and bacterial proteases. The antimicrobials dissolved in zymography development media exerted antiprotease effects on both eukaryotic and bacterial proteases (Fig 1). For bacterial proteases, PVP-I and octenidine achieved statistically significant (p<0.05) reductions in protease activity at the highest tested concentrations. Silver lactate treatment resulted in the significantly (p<0.05) decreased activity of bacterial proteases in the two more diluted samples, while there was surprisingly high variability in the inhibition of protease activity in the least diluted silver lactate solution. The activity of bacterial proteases correlated inversely with chlorhexidine and octenidine concentrations. Among all tested conditions, PVP-I at the highest concentration inhibited bacterial proteases most potently (49% of the activity of control); however, the dilution of PVP-I decreased the antiprotease effect in a non-linear fashion.

We observed that the antimicrobials inhibited eukaryotic proteases even more than bacterial proteases. Also, the activity of human proteases was inhibited dose-dependently, similarly to bacterial proteases. As a result, silver lactate and octenidine hydrochloride significantly (p<0.05) inhibited eukaryotic proteases at all tested concentrations. Again, PVP-I at the highest concentration decreased the activity of eukaryotic proteases to 1%, but diluted PVP-I did not significantly inhibit proteases.

**Fig 2:**
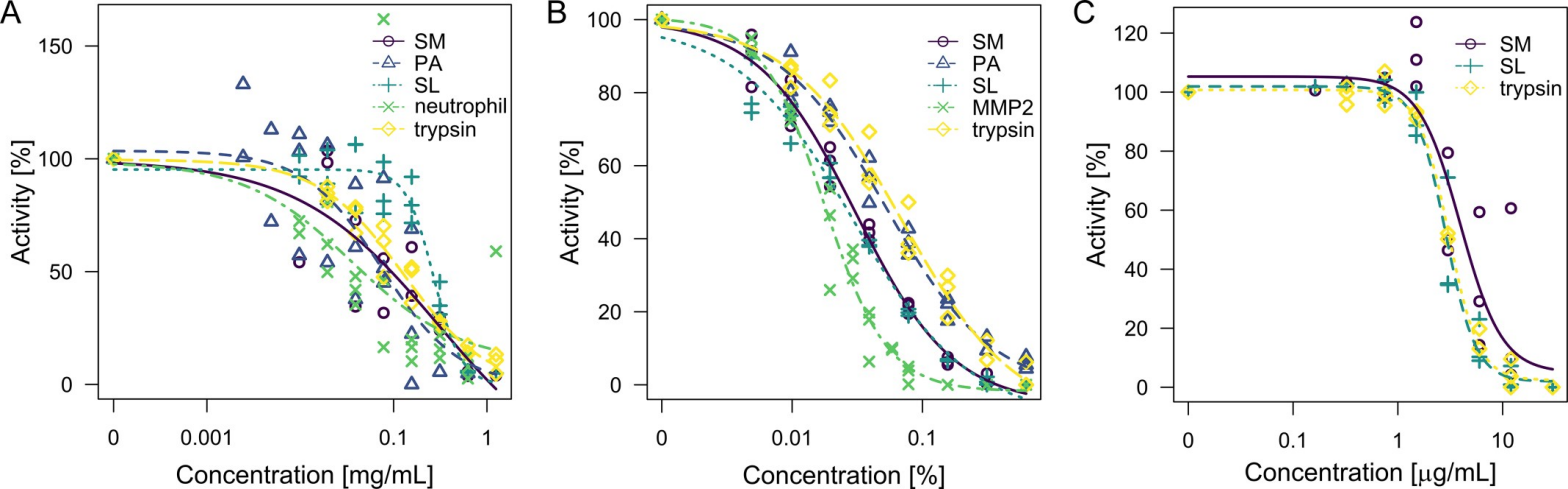
Activity of bacterial proteases with antimicrobials in fluorogenic assay. Pure proteases (MMP-2 or trypsin) or single-source proteases from bacterial or eukaryotic media were evaluated in fluorogenic assay with quenched gelatin as a substrate. Reactions were in the presence or absence of different concentrations of silver lactate (A), chlorhexidine digluconate (B), and octenidine hydrochloride (C). Differences in fluorescence increase were related to control without antiseptics. Measurements were repeated two to three times. X-axis is in log10 scale. PA–*P*. *aeruginosa*, SL–*S*. *liquefaciens*, SM—*S*. *marcescens*.

To gain a better insight into the inhibition process, we further evaluated single sources of proteases in a fluorogenic assay, which increased sensitivity and allowed continuous measurements (Fig 2). We also included in these experiments trypsin, which is a standard protease with robust activity. However, some proteases were not active under the fluorogenic assay conditions and therefore were not measured. In addition, this assay was not suitable for iodine as it quenches fluorescence. Despite the drawbacks, the results again showed that chlorhexidine and octenidine inhibited proteases in a dose-dependent manner. Silver lactate also induced a dose-dependent response–however, with greater deviations. The IC50 for the evaluated proteases were in the range 0.02–0.07% for chlorhexidine, 2.87–3.95 μg.mL^-1^ for octenidine, and 0.04–0.30 mg.mL^-1^ for silver lactate (S1 Table). Where MMP-2 and leukocyte proteases were active, IC50 was the lowest from the evaluated proteases, which supports the results obtained with SDS-PAGE gelatin zymography.

**Fig 3:**
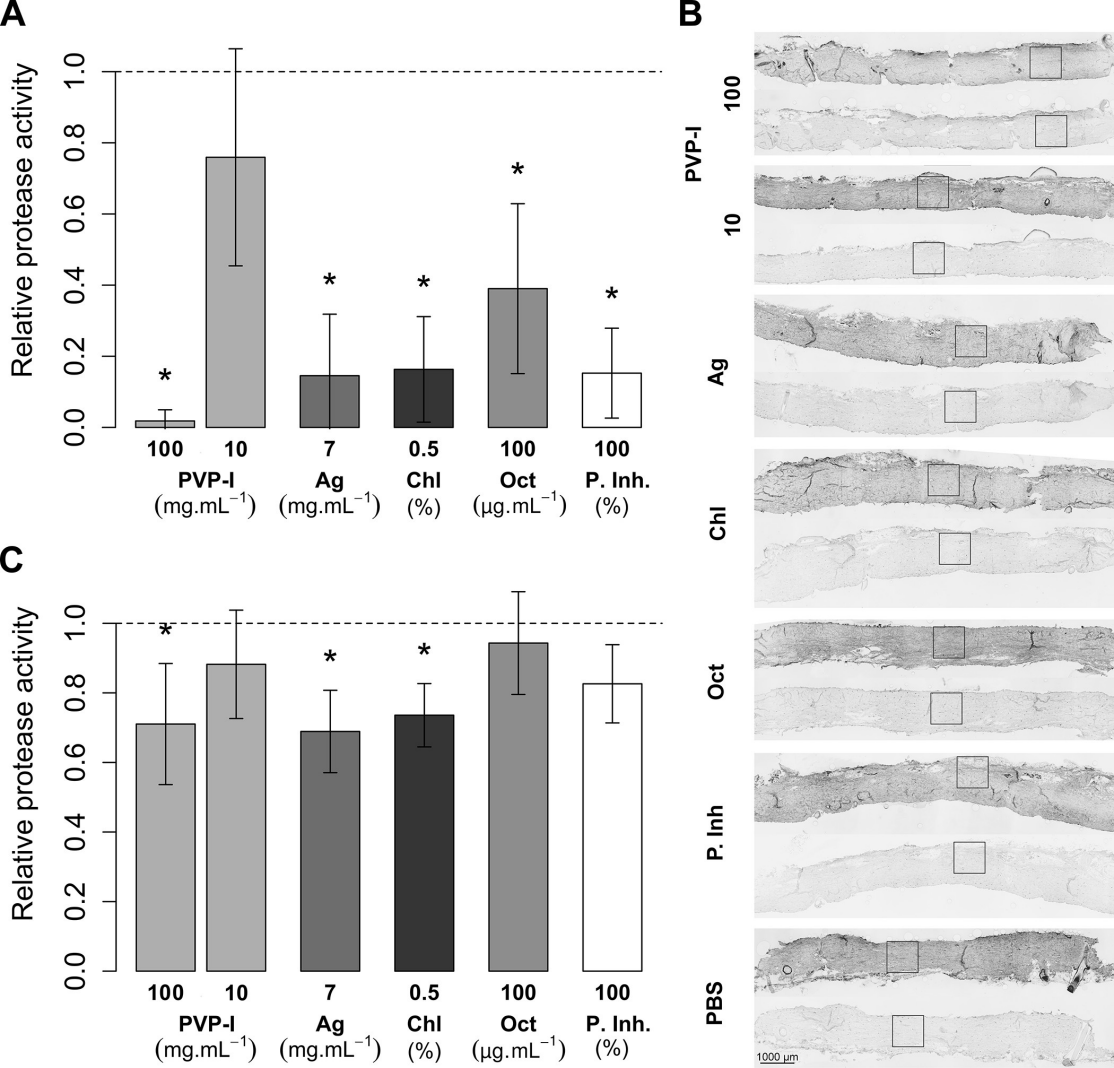
Activity of porcine skin endogenous proteases with antimicrobials. Whole skin was incubated with the tested compounds in a tube, homogenized, and assessed by means of gelatin zymography (A) or incubated in Franz diffusion cells (dermal side up) and analysed using *in situ* zymography, as illustrated with representative images (B). Color-inverted images, darker shades correspond to increased proteolysis. Each sample was developed with fluorogenic substrate either at 37°C (the upper sample section of each treatment) or at -20°C (assay control, the lower sample section). Squares show areas of fluorescence intensity measurement, which were evaluated in replicates (n = 5) with image analysis (C). Bars in (A) and (C) show mean protease activity related to PBS control ± SD. * denotes p < 0.05 (Student's one sample t-test to control). The tested compounds were povidone-iodine (PVP-I), silver lactate (Ag), chlorhexidine digluconate (Chl), octenidine hydrochloride (Oct), commercial protease inhibitors (P. Inh.).

To extend the observations that antiseptics were able to inhibit proteases in polyacrylamide gels and in solution, we investigated whether antiseptics could reach and inhibit proteases in porcine dermis. The dermis was incubated with the tested compounds in a tube and then homogenized and assessed using gelatin zymography (Fig 3). We found that protease activity was significantly (p<0.05) diminished after treatment with silver lactate, chlorhexidine, octenidine, and a commercial protease inhibitor cocktail at the highest concentrations as used for protease inhibition in polyacrylamide gels. However, to inhibit proteases in skin, the PVP-I concentration had to be higher than that required to inhibit proteases in polyacrylamide gel.

Porcine skin samples were incubated with antiseptics in a donor fluid in Franz diffusion cells to simulate more accurately open wounds, where antiseptics may be in contact only with the wound bed. We investigated protease activity with fluorescent *in situ* zymography (Fig 2). All the tested antimicrobials showed a tendency to decrease protease activity; however, the decrease was significant (p<0.05) only in 10% PVP-I, silver lactate and chlorhexidine. Commercial protease inhibitors also decreased proteolytic activity, although not significantly (p>0.05).

## Discussion

We found that PVP-I, silver lactate, chlorhexidine digluconate, and octenidine hydrochloride exhibited antiprotease activities *in vitro* and *ex vivo*. The inhibitory effect was dose-dependent and more pronounced on eukaryotic proteases.

Iodine released from povidone iodine was reported to inhibit the activity of human neutrophil elastase, plasmin, and matrix metalloproteases as well as the proteolytic activity of exudates from human chronic wounds in a dose-dependent manner [5]. Also, iodine-releasing bandages were able to inhibit collagenases or papain [6]. Our results confirm these observations and broaden the list of proteases inhibited by iodine for the tested wound bacteria. The usual concentration of povidone-iodine for external use is 5–10% and our results show that even 1% povidone-iodine is sufficient to inhibit bacterial and eukaryotic proteases; however, 10% PVP-I was needed to inhibit proteases in skin. Iodine oxidizes several amino acids by covalent modification and thus disrupts the activity of enzymes or even precipitates them [17]. Iodine stability and evaporation is an issue and this is also demonstrated in our results, where less diluted PVP-I readily inhibited proteases. The effect, however, was variable in higher dilutions of PVP-I. This may be a target for optimization in future advanced medical devices that release iodine.

Silver in wound devices is in the form of silver salts (e.g. AgNO_3_), nanoparticles, or colloidal silver. Still, all forms need to be converted into ionic silver to exhibit antimicrobial properties [18]. Different silver forms mainly vary in the release of ionic silver and thus in their bioavailability with respect to possibly inhibiting proteases. Silver nitrate in colorimetric assay inhibited MMP-2, MMP-8, and MMP-9 by more than 90%, although in much higher concentrations than the silver lactate that we used [9]. A study comparing the inhibition of the debriding enzymes papain and *Clostridium* collagenase by means of antimicrobial dressings showed that the bandage with nanocrystalline silver inhibited both the activity of collagenase (by 52%) and papain (by 12%), which was less than the two tested iodine-releasing devices [6]. Silver inhibited trypsin and chymotrypsin reversibly and the effect was annulled when Cl^-^ ions were present and AgCl precipitate formed [19]. Therefore, we replaced Tris-HCl buffer for zymography development with HEPES buffer and incubated porcine skin in a water solution of silver lactate. Also, we desalted DQ gelatin substrate and proteases with buffers without chloride salts or with water. It remains open how the release of Ag^+^ from various forms would affect its bioavailability to inhibit proteases and its capacity to avoid Ag^+^ precipitation.

Chlorhexidine is known to inhibit matrix metalloproteinases [8] and bacterial proteases in dental plaque [7]. Effective chlorhexidine concentrations *in vitro* may vary from 0.0001% for MMP-2 to > 0.02% for activated MMP-8 [8] and also depend on the bacterial strain [7]. In this study and in a similar experimental setup, chlorhexidine digluconate was not able to fully inhibit eukaryotic proteases even at higher concentrations when assessed with SDS-PAGE zymography. However, chlorhexidine inhibited MMP-2 at lower concentrations (IC50 0.02%) in fluorogenic assay. Several staphylococcal proteases were inhibited by chlorhexidine [7]. We have found no published evidence of the inhibition of *Pseudomonas aeruginosa*, *Serratia marcescens* or *Serratia liquefaciens* proteases by chlorhexidine. Our results thus extend the list of proteases inhibited by chlorhexidine with respect to those relevant to wound healing. The usual concentration of chlorhexidine required for wound irrigation is 0.1%, which inhibited eukaryotic but not prokaryotic proteases in SDS-PAGE zymography and did not fully inhibit either eukaryotic or prokaryotic proteases in fluorogenic assays. Plausibly, chlorhexidine action could include nonspecific antiprotease mechanisms that could either transiently or permanently inhibit proteases. Such a reversible protease-inhibiting property may be attributed to the ability of chlorhexidine to chelate calcium ions [8], which were, however, in excess in our zymographic buffers for chlorhexidine. More likely, the short-term incubation that led to protein precipitation was responsible for protease inhibition [20].

To our knowledge, the antiprotease activity of octenidine was not previously reported. Our results suggest clear dose-dependent inhibitory action on bacterial as well as human proteases in the concentrations tested (100, 20, 4) with even lower IC50 (3.07 μg.mL^-1^ for trypsin) determined from fluorogenic assay. A commercially available wound irrigation solution contains 1 000 μg.mL^-1^ octenidine dihydrochloride and it is therefore possible that the solution may inhibit *in situ* chronic wound proteases. Octenidine exerts its antimicrobial effects due to positively charged centers and hydrophobic chains, which readily disrupt the bacterial cell wall [21].

Despite the protease-inhibiting activity of the antimicrobials demonstrated here and in published literature, the effects may not be so straightforward in the complex environment of chronic wounds. First, it is not clear what the ideal wound depth should be, i.e., the depth at which antimicrobials should most effectively modulate host protease activity. Only a few studies have investigated proteolytic activity deeper in chronic wound tissue, where proteases are shielded from direct contact with antimicrobials, as the majority of studies focus on wound exudate [2,22]. We addressed this by showing that chlorhexidine digluconate and silver lactate can penetrate skin and inhibit proteases *in situ* when in full contact with skin or even in partial contact in Franz diffusion cells. Although PVP iodine potently inhibited proteases, its ability to penetrate skin was lower when diluted. Also, octenidine hydrochloride inhibited skin proteases when skin was submerged in its solution; however, this effect was not significant (p>0.05) in Franz diffusion cell experiments, which corresponds to the previously reported low absorption of octenidine dihydrochloride through skin [23]. The ability to penetrate deeper into the wound may be affected by the kinetics of release from the particular device and the actual delivery system of the antimicrobial. This may affect penetration in the case of iodine, which evaporates rather quickly, or the amount of free Ag^+^ that precipitates with Cl^-^. Second, there are potentially negative effects of antimicrobials or restrictions on their use. Due to nonspecific effects on proteins, antimicrobials may modify growth factors and cytokines and potentially disrupt their biological activity in chronic as well as acute wounds. This raises the question of whether the protease-modulating activity of antimicrobials would aid chronic wound healing despite their possible shortcomings. A recent *in vivo* study correlated faster wound healing with the absorption of proteases into a bandage of regenerated cellulose and showed that even a superficial decrease in protease activity may aid wound healing [4].

Overall, we have shown that povidone-iodine, silver lactate, chlorhexidine digluconate, and, for the first time, also octenidine hydrochloride inhibited wound bacterial and eukaryotic proteases. Also, these antiseptics had varying abilities to penetrate into skin and to inhibit resident proteases. Although these results need to be verified *in vivo*, there is a strong possibility that by choosing a suitable antibacterial treatment, one may also address elevated wound proteases. Future advanced wound bandages may therefore be designed to optimize both the antimicrobial and antiprotease effects of antiseptics.

## Supporting information

S1 Table Inhibitory concentrations (50%) gained from dose-response curves from fluorogenic assays with varying concentrations of the antiseptics. PA–P. aeruginosa, SL–S. liquefaciens, SM—S. marcescens.
(DOCX)
