# Supplementary material for: Dual role of iodine, silver, chlorhexidine and octenidine as antimicrobial and antiprotease agents
Source: PLoS One. 2019 Jan 31;14(1):e0211055. doi: 10.1371/journal.pone.0211055 (PMC6355201; doi:10.1371/journal.pone.0211055)
Supplement: S1 Table — PA–P. aeruginosa, SL–S. liquefaciens, SM—S. marcescens. (DOCX) [file pone.0211055.s001.docx]

| Antiseptic | Protease source | |  |  |  |
| --- | --- | --- | --- | --- | --- |
|  |  | IC50 [mg/mL] | SEM | Lower 95 CI | Upper 95 CI |
| silver lactate | neutrophil | 0.04 | 0.03 | -0.02 | 0.09 |
|  | PA | 0.08 | 0.05 | -0.02 | 0.17 |
|  | SL | 0.27 | 0.06 | 0.16 | 0.38 |
|  | SM | 0.30 | 1.18 | -2.04 | 2.64 |
|  | trypsin | 0.13 | 0.09 | -0.05 | 0.31 |
| chlorhexidine digluconate |  | IC50 [%] | SEM | Lower 95 CI | Upper 95 CI |
|  | MMP-2 | 0.02 | 0.00 | 0.02 | 0.02 |
|  | PA | 0.05 | 0.01 | 0.04 | 0.06 |
|  | SK | 0.03 | 0.00 | 0.02 | 0.04 |
|  | SM | 0.03 | 0.00 | 0.02 | 0.04 |
|  | trypsin | 0.07 | 0.01 | 0.05 | 0.09 |
| octenidine dihydrochloride |  | IC50 [ug/mL] | SEM | Lower 95 CI | Upper 95 CI |
|  | SL | 2.87 | 0.27 | 2.33 | 3.41 |
|  | SM | 3.95 | 0.65 | 2.64 | 5.26 |
|  | trypsin | 3.07 | 0.35 | 2.37 | 3.77 |
